# Supplementary material for: Susceptibility to acute cognitive dysfunction in aged mice is underpinned by reduced white matter integrity and microgliosis
Source: Commun Biol. 2024 Jan 16;7:105. doi: 10.1038/s42003-023-05662-9 (PMC10791665; doi:10.1038/s42003-023-05662-9)
Supplement: Supplementary file 2 — Supplementary material [file 42003_2023_5662_MOESM2_ESM.pdf]

Supplementary Material

Sickness behaviour recovery over 24 hours

Aged animals (20-24 months) treated with LPS showed more severe and prolonged sickness responses than adult mice (<10 months). In this cohort, this pattern is typically more obvious at 24 hours than at 6 hours. Temperature is modestly more affected by LPS in older animals at 6 hours and significantly so at 24 hours. Rears are very markedly more suppressed by LPS in older mice than in younger, whether observing at 6 or 24 hours. Distance travelled was suppressed equally in older and younger mice at 6 hours post-LPS but the effect persists at 24 hours in old mice while younger animals show more recovery of towards their baseline performance. Statistical analysis is as described in the figure legend.

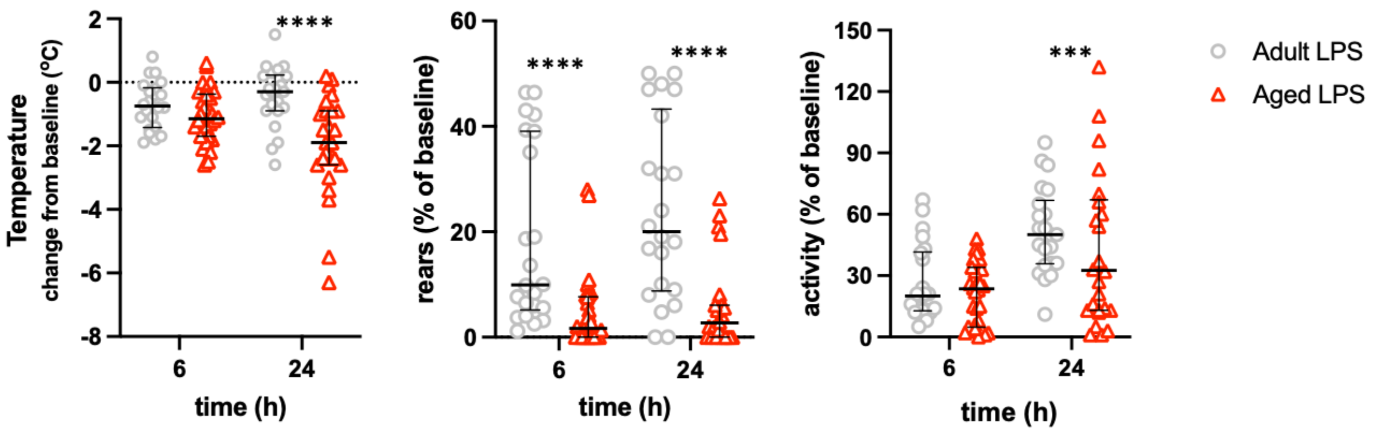

Supplementary figure 1: LPS induces exaggerated sickness responses in aged mice between 6 and 24 hours. Effect of age on sickness behaviour response to LPS (100µg/kg i.p.) measured at 6 and 24 hours-post intraperitoneal LPS. Sickness was measured as A) change from baseline core-body hypothermia, B) percentage change from baseline rearing behavior in the open field and C) percentage change from baseline locomotor activity (distance covered) in the open field. Data were non-parametric and are presented as median ± interquartile range and were analysed by Kruskal Wallis followed, upon significant main effects, by Dunn multiple comparisons test (n = 22 for adult mice and 30 for aged mice). Stars denote significant difference between young and aged responses to LPS treatment: * (p<0.05),**(p<0.01), ***(p<0.001), ****(p<0.0001).

LPS-induced Pro-inflammatory cytokines expression across the aged brain

Transcription of the genes for the proinflammatory cytokines IL-1 alpha, IL-1beta and TNF-alpha was induced by LPS (100 µg/Kg) in all 4 brain regions assessed. However, induction was typically exaggerated in aged animals with respect to younger adults. This was true for *Il1a* and *Il1b* in all regions examined and, for *Tnfa*, in all regions except the hippocampus.

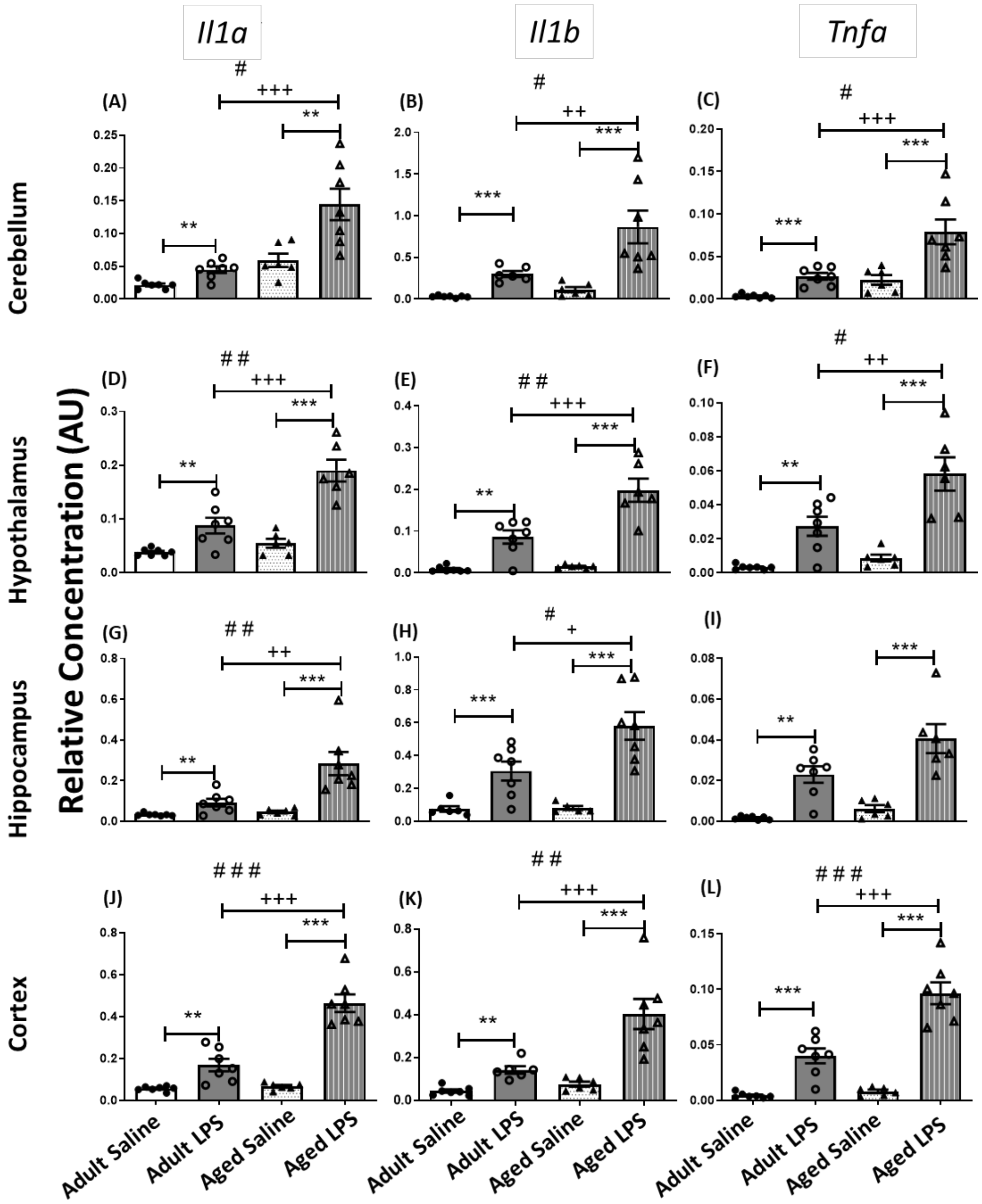

Supplementary Figure 2: LPS-induced Pro-inflammatory cytokines expression across the aged brain: Effect of age on pro-inflammatory cytokine gene transcript expression in cerebellum, hypothalamus, hippocampus and prefrontal cortex 4 hours post LPS (100 µg/kg) i.p. challenge. All data represented by mean ± SEM (n=6,7) and analysed by two-way ANOVA followed by a Bonferroni post-hoc test; * denotes a statistically significant effect of LPS treatment, * ($p<0.05$), ** ($p<0.01$), *** ($p<0.001$). # denotes a statistically significant interaction between age and treatment; # ($p<0.05$), ## ($p<0.01$), ### ($p<0.001$). + denotes a statistically significant effect of age on response to LPS treatment; + ($p<0.05$), ++ ($p<0.01$), +++ ($p<0.001$).

Figure 3 Sex-dependent differences in cognitive function.

Saline had trivial effects on working memory function at all ages, in both sexes, with performance varying modestly either side of 80%. LPS had a more robust effect on male animals than female animals at 16-19 months, while having similar effects in both sexes at 5-7 months and at 24 months. There was a main effect of sex ($F_{1,10}$=6.21; p=0.031), while the interaction of sex and time post-challenge was not quite significant ($F_{14,140}$=1.5; P=0.118). There was a significant pair-wise comparison at 75 hours (i.e. the first block after LPS treatment, p=0.0138). Baseline working memory function was lower in males before LPS challenge, which may influence increased vulnerability to disruption at that age.

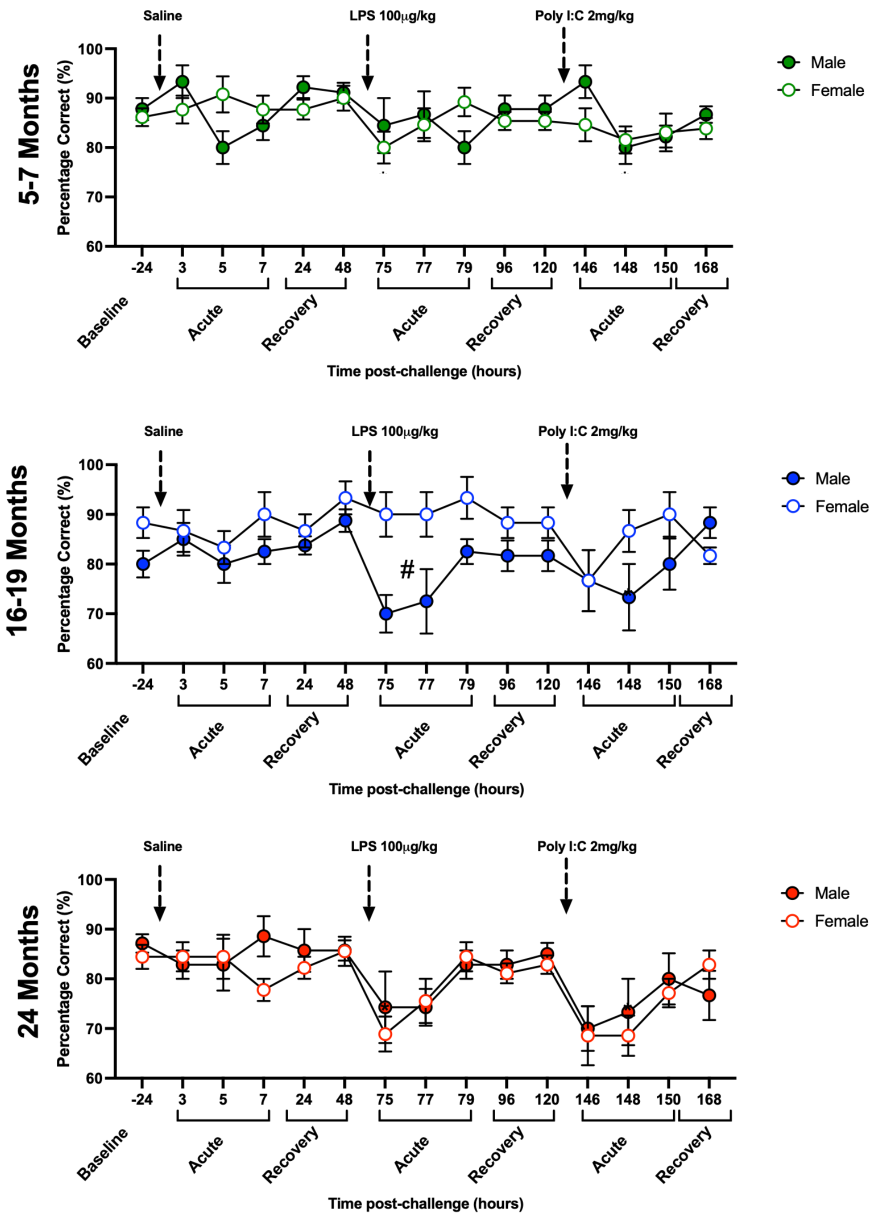

Supplementary Figure 3. Sex-dependence of cognitive vulnerability to systemic inflammation-induced acute cognitive deficits. Impact of consecutive saline, LPS (100 µg/kg i.p.) and Poly I:C (12 mg/kg i.p.) on working memory assessed by T-Maze. All data are represented graphically by mean ± SEM (24 months n=13, 16-19 months n=12, 5-7 months n=22), analysed by repeated measures two-way ANOVA, across the full time course with multiple comparisons by Bonferroni post-hoc test, # denotes a significant different between male and female immediately after LPS treatment (p=0.0138).

Figure 4 Region-dependent differences in number of microglial cells

Results for IBA-1, reported in the main results text, were corroborated by a significant increase in the microglial specific nuclear marker Pu.1 in the same 3 regions (fimbria p<0.001 = $F_{2,14}$ = 17.43; CC p<0.001, $F_{2,13}$= 16.6,; DN = p<0.001 non-parametric). There were no clear differences in microglial number/activation between young and cognitively resilient older animals.

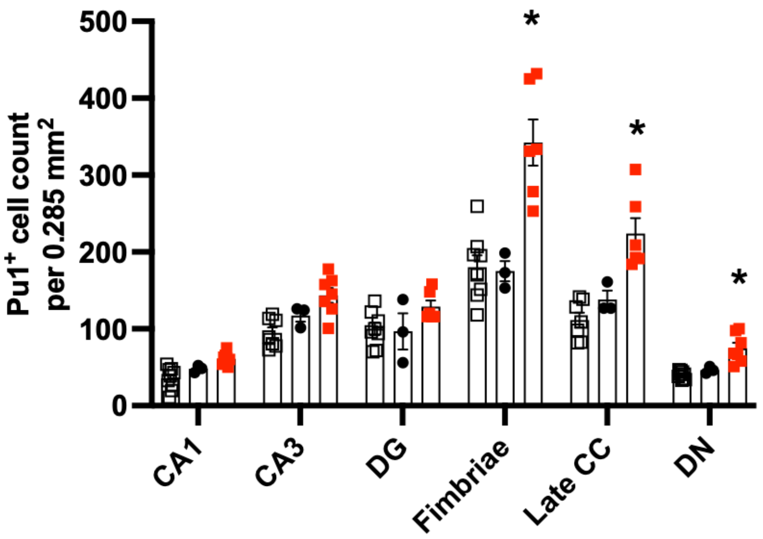

Supplementary figure 4: Relationship between cognitive status and quantitative analysis of microglial numbers in different brain regions. Quantification of positively stained Pu1+ microglial cells in multiple areas of the hippocampal formation, categorised by age & cognitive frailty; quantified using Image J (NIH) at 20x. Each animal is represented by an individual data point and these non-parametric data were analysed by Kruskal-Wallis with Dunn's post-hoc tests to detect microglial differences between young, (n=10), old cognitively resilient (n=3) and old cognitively frail (n=7) Statistically significant post-hoc differences between aged cognitively resilient and aged cognitively frail animals after significant main effects are denoted by *p<0.05, **p<0.01.
