# Supplementary material for: Susceptibility to acute cognitive dysfunction in aged mice is underpinned by reduced white matter integrity and microgliosis
Source: Commun Biol. 2024 Jan 16;7:105. doi: 10.1038/s42003-023-05662-9 (PMC10791665; doi:10.1038/s42003-023-05662-9)
Supplement: Supplementary file 3 — Description of Supplementary Materials [file 42003_2023_5662_MOESM3_ESM.docx]

**Description of Additional Supplementary Files**

**File name:** Supplementary Data 1

**Description:** This excel file contains all of the data that populate the graphs in all figures.
